# P311, Friend, or Foe of Tissue Fibrosis?

**DOI:** 10.3389/fphar.2018.01151

**Published:** 2018-10-12

**Authors:** Leslie Stradiot, Inge Mannaerts, Leo A. van Grunsven

**Affiliations:** Liver Cell Biology Lab, Vrije Universiteit Brussel, Brussels, Belgium

**Keywords:** P311, fibrosis, proliferation, migration, TGFβ1

## Abstract

P311 was first identified by the group of Studler et al. (1993) in the developing brain. In healthy, but mainly in pathological tissues, P311 is implicated in cell migration and proliferation. Furthermore, evidence in models of tissue fibrosis points to the colocalization with and the stimulation of transforming growth factor β1 by P311. This review provides a comprehensive overview on P311 and discusses its potential as an anti-fibrotic target.

## Introduction

Neuronal protein 3.1 (P311) is a small intracellular protein from an unknown family, whose transcript was first identified by Studler et al. (1993) in a differential screening comparing striatal cells from two different stages of brain development. P311 knockout mice have learning and memory deficiency and a disturbed pain affection (Sun et al., 2008; Taylor et al., 2008). During the past years, light has been shed on the broader expression pattern of P311 and its function in both health and pathology. P311 has proven to be a multifunctional protein with important implications in development, disease, and regeneration. For this review, we explore P311 functions and discuss possible implications of P311 in the development of tissue fibrosis by highlighting its role in tissue regeneration, cell migration, and its interaction with the pro-fibrotic cytokines transforming growth factor β1 and 2 (TGFβ1 and 2).

## General Concepts in Tissue Fibrosis

**FIGURE 1:**
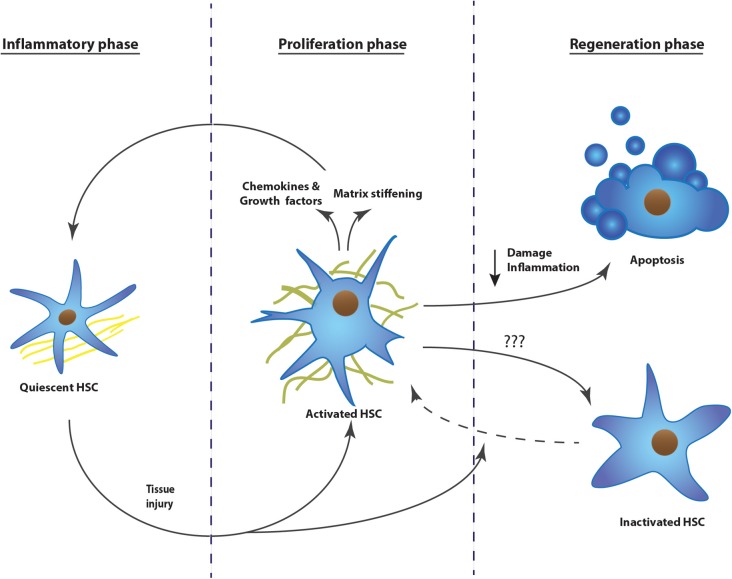
The development of tissue fibrosis progresses in three phases: inflammatory phase, proliferation phase, and regeneration phase. Upon repeated injury, the process continuously repeats phase one and two, without resolution of the scar tissue. The example represents the activation mechanism of hepatic stellate cells during the development of liver fibrosis. HSC, hepatic stellate cell.

Tissue fibrosis can be defined as the hardening of epithelial tissues due to accumulation of secreted collagens. Initially it starts as a wound healing response, but upon repeated insults, the scarring increases and becomes pathogenic (Friedman et al., 2013). Under normal conditions, wound healing undergoes three phases; an inflammatory, a proliferative, and a regenerative phase. These phases are well characterized for liver fibrosis (**Figure 1**), while lung and kidney present a similar fibrotic process (Friedman et al., 2013).

### Inflammatory Phase

Upon tissue injury, we can distinguish different origins of pro-fibrogenic stimuli. Damaged endothelium and epithelium as well as activated platelets will secrete pro-fibrogenic and pro-inflammatory cytokines. Furthermore, they also attract extra mesenchymal cells, inter-organ and organ resident cells to the site of injury by secreting chemokines (Dranoff et al., 2004; Armulik et al., 2005; Darby et al., 2014).

### Proliferative Phase

Due to the inflammatory environment, fibroblasts will activate and differentiate into myofibroblasts. These will proliferate, gain tensile forces, and will secrete chemokines to attract other fibroblasts. Myofibroblasts at the site of injury will secrete a massive amount of matrix proteins, replacing the fibrin matrix with a dense collagen-rich one (Darby et al., 2014; Simone et al., 2015).

### Regeneration Phase

Once the insult is ceased, damage and inflammation is diminished and collagens will be degraded, mainly by matrix metalloproteinases. This will allow re-epithelialization. Most of the myofibroblasts present undergo apoptosis during healing or revert to an inactive phenotype as was shown in the liver (Desmouliere et al., 1995; Kisseleva et al., 2012; Darby et al., 2014).

When the injury is chronic, the first two phases are continuously repeated and the matrix resolution and epithelial restoration do not occur. The fibrotic site will expand, the organ tissue will continue to harden, leading to cellular dysfunction and eventually organ failure (**Figure 1**). This pathological process has been described in several organs, but is most intensely studied in liver, kidney, and lung. Hepatic stellate cells, renal mesangial cells, and resident lung fibroblasts, respectively, are the organ-specific resident cells that transdifferentiate to myofibroblasts and contribute to the scarring (Ardaillou et al., 1999; Friedman, 2008; Barkauskas and Noble, 2014). During the proliferative phase, activation of these cells is triggered by different pathways; damaged epithelial cells will stimulate fibroblast activation either by their released nucleotides or by expressing integrins, which will then mechanically activate TGFβ1 that is transiently stored in the extracellular matrix (Munger et al., 1999; Dranoff et al., 2004; Zhan et al., 2006; Giacomini et al., 2012). Platelets present at the site of injury will recruit fibroblasts as well as inflammatory cells, which secrete pro-fibrogenic cytokines like tumor necrosis factor α, interleukin (IL) 1β, IL13, and IL17 (Friedman et al., 2013; Darby et al., 2014). Upon activation, the myofibroblasts themselves will secrete chemokines, cytokines, as well as oxygen radicals, leading again to increased stress, immune response, and myofibroblast activation (Friedman et al., 2013). Once fibrosis is initiated, it is a self-stimulating system; matrix-stored TGFβ1 is activated by the epithelial cells, this active TGFβ1 in turn activates fibroblasts, which will then recruit and activate even more fibroblasts, which will produce TGFβ1 and so on (Ignotz and Massague, 1987; Wang et al., 1996; Giacomini et al., 2012). In parallel, more myofibroblasts means more collagen secretion and crosslinking, finally leading to stiffening of the extracellular matrix. A stiff matrix in turn stimulates proliferation, drives cells to activate, inhibits the production of matrix digestive enzymes, and facilitates the activation of TGFβ1 in the matrix (Li et al., 2007, Liu et al., 2010; Olsen et al., 2011; Huang et al., 2012).

The organ resident stromal cells are important targets to attenuate fibrosis development. Interfering with their recruitment, proliferation, TGFβ1 response, or collagen secretion already greatly delays or sometimes even stops fibrosis progression in animal models of fibrosis (Munger et al., 1999; Liu et al., 2009a; Kuroda et al., 2015; Nayak et al., 2016). Unfortunately, these approaches have not yet evolved in clinically approved therapeutics (Schuppan and Kim, 2013; Schon et al., 2016). P311 is involved in several of these fibrosis stimulating pathways and might represent an interesting target for the development of anti-fibrotic drugs. We will discuss its involvement in cell migration, regeneration, and development in different tissues.

## The Basics of P311

The first studies on P311 reported the transcript in the germinal zones of the brain at embryonic day 17, the transcript was observed in the striatum and the superficial cortical layers at E20, suggesting that the gene was expressed in cells from the second germinal migration wave. The expression persists during adulthood, where it is present in several brain regions with active postnatal neurogenesis like the cerebellum, hippocampus, and the olfactory bulb (Studler et al., 1993). P311 has been alternatively named pentylenetetrazole 17, since it potentiates an inward calcium current upon pentylenetetrazole administration in neurons, mimicking epileptic bursting (Kajiwara et al., 1995). The P311 open reading frame is located on chromosome 18 in mice, on chromosome 5 in humans, and has been highly conserved among mice, humans, rhesus monkeys, dogs, etc. This sequence is translated into an 8 kDA protein of 68 amino acids and contains a PEST domain responsible for the fast degradation by ubiquitin-dependent degradation as well as by an unknown metalloprotease, resulting in a half-life of approximately 5 min (Studler et al., 1993; Taylor et al., 2000). Research for P311 binding partners, using a predictor of naturally disordered regions analysis, suggested that P311 is an intrinsically disordered protein that needs an interaction partner to acquire a tertiary structure. Indeed, P311 was shown to interact with several cytoskeletal proteins such as Filamin A and non-muscle myosin heavy chain 9 (MYH9) and with eukaryotic translation initiation factor 3, subunit B (eIF3b), a component of the translation initiation complex (Yue et al., 2014).

## P311 Is Expressed in Migrating Cells and Stimulates the RAC1 Pathway

Fibroblast migration is a key event for organ development as well as fibrosis (Shimizu et al., 2001; Hattori et al., 2004; Scholten et al., 2011). P311 is described by different groups to play an important role during chemokine-induced cell migration (Studler et al., 1993; Mariani et al., 2001; McDonough et al., 2005; Guimaraes et al., 2015). The first study ever describing P311 already detected the mRNA in migrating cells in the developing brain (Studler et al., 1993). A study aiming at discovering mechanisms involved in invading glioma cells, determined that P311 is highly expressed in the invasive rim of human glioma tumors, when compared to P311 expression in the tumor core (∼threefold). Furthermore, knockdown of P311 in human glioma cells (SF767) reduced cell migration *in vitro*, while when cultured on glioma-derived extracellular membrane, glioma cells migrated at a higher rate and expressed higher P311 levels (Mariani et al., 2001). This correlation between P311 and cell migration was also shown during liver fibrosis. Upon liver injury, hepatic stellate cells activate, resulting in contractile and migrating hepatic stellate cells with increased P311 transcription (Guimaraes et al., 2015). When activated chronically, these cells deposit high levels of collagen, resulting in liver fibrosis and finally cirrhosis. Knocking down P311 expression in cultured primary hepatic stellate cells reduced chemokine-dependent cell migration (Guimaraes et al., 2015). Furthermore, immunohistochemical stainings demonstrated that the P311 protein was present in nuclei as well as at leading edges of migrating hepatic stellate cells and human astrocytes (Taylor et al., 2000; Guimaraes et al., 2015). In hepatic stellate cells and glioma cells, less P311 expression also resulted in less lamellipodia, most likely resulting in a reduced migration (Mariani et al., 2001; Guimaraes et al., 2015).

**FIGURE 2:**
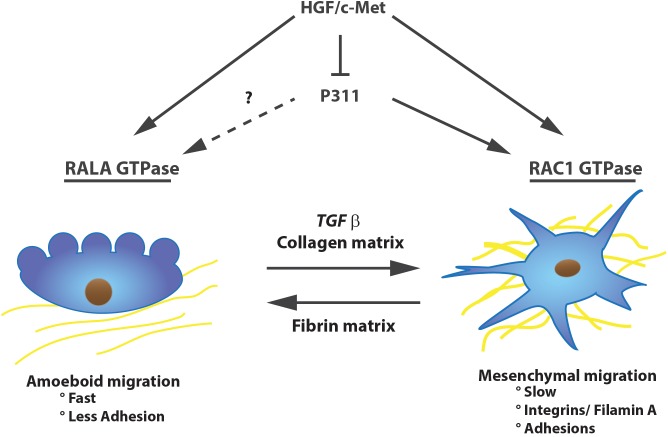
P311 is expressed in migrating cells. P311 can induce RAC1 GTPase to induce slow mesenchymal migration, whether it stimulates RALA GTPase, inducer of amoeboidal migration, is unknown. Nevertheless, independent of the migration type, P311 is expressed in migrating cells. Upon stimulation with HGF/c-Met, cells will initiate migration but they will downregulate P311 expression.

P311-stimulated migration is strongly dependent on the half-life of the protein, which is regulated by constitutive phosphorylation of a serine (Ser59, situated right next to the PEST domain) by protein kinase C δ, ε, and ζ, resulting in a short-lived P311 protein and no migration. β1 integrin signaling on the other hand stimulates its dephosphorylation and consequently cell migration. Upon plating U118 cells on a motility-activating substrate Ser59’s phosphorylation is reduced, P311 is not degraded and indirectly stimulates ras-related C3 botulinum toxin substrate 1 (RAC1) GTPase activity (McDonough et al., 2005). Additionally, mice with RAC1-deficient fibroblasts have impaired cutaneous wound healing and the fibroblasts have a less activated phenotype (Liu et al., 2009b). RAC1 GTPase was also shown to be important for glioma cell migration and is highly expressed in cells performing mesenchymal migration (Shaw et al., 1997; Huang et al., 2014). This type of migration acts “slow” and occurs with a lot of adhesions and integrin/filaminA interactions. On the other hand, amoeboid migration is faster and is characterized by less adhesions and mainly uses Ras-related protein A (RALA) and Ras homolog gene family, member A (RHOA) GTPases instead of RAC1 (**Figure 2**). P311 does not seem to be specific for one or the other migration mechanism. Fibroblasts overexpressing P311 can migrate in both fashions, depending on the culture dish coating or cytokine treatment, such as TGFβ1 (Shi et al., 2006; Huang et al., 2014). Correspondingly, human epidermal stem cells initiate P311 expression in response to skin damage to promote wound re-epithelialization. This was shown *in vitro* in human epidermal stem cells in which overexpression of P311 resulted in a faster migration through stimulation of RHOA and RAC1 activity while in P311 knockout mice skin wounds healed slower (Yao et al., 2017).

**FIGURE 3:**
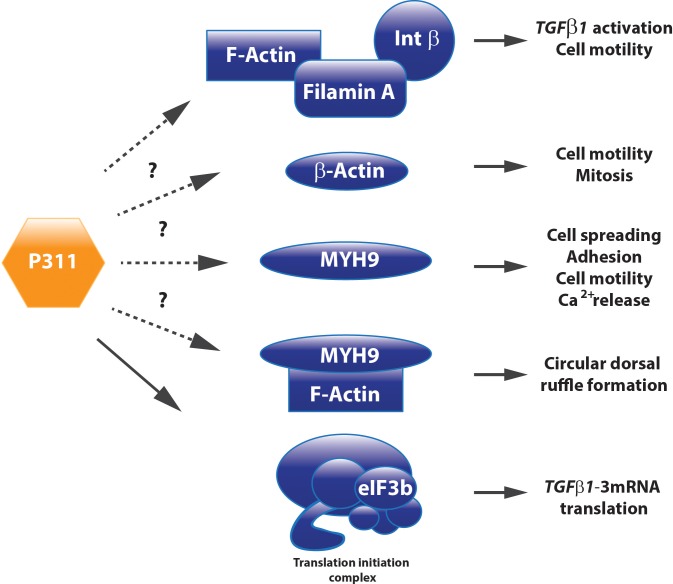
P311 has different interacting proteins. Co-immune precipitation analysis determined Filamin A, MYH9, Actin ß, and eIF3b as P311 interacting proteins. Whether they need P311 to correctly perform their function still remains uncertain for most of these binding partners.

Filamin A, an interconnecting protein between F-actin and β1 integrin binding protein, was identified as a direct binding protein of P311 in glioma cells. Later on, this was confirmed in 3T3 cells overexpressing a Myc-tagged P311 protein. Co-immune precipitation and mass spectrometric analysis demonstrated that P311 interacts with cytoskeletal proteins MYH9, actin β, and filamin A (**Figure 3**). Filamin A was shown to interact with integrin β1, present in the cell membrane, to activate TGFβ1 and regulate cell motility (McDonough et al., 2005). Actin β is a fundamental cytoskeleton protein that is one of the driving forces for cell protrusions (Bunnell et al., 2011). MYH9 binds transiently to the cytoskeleton, by which it regulates cell spreading, adhesion, and migration (Huang et al., 2009; Liu et al., 2011; Casalou et al., 2014). One study on hepatic stellate cells demonstrated that MYH9 enabled intracellular Ca^2+^ release, a feature that was also described to P311 when administered to neuronal cells (Kajiwara et al., 1995; Liu et al., 2011). Together with F-actin, MYH9 is also involved in the formation of circular dorsal ruffles upon PDGF-BB stimulation, which recycle integrins, remodel the cytoskeleton during migration, and their presence is enhanced by RAC1 (Casalou et al., 2014; **Figure 3**). Further research regarding the role of P311’s interaction with these two proteins and the potential effect on circular dorsal ruffle formation still needs to be done (McDonough et al., 2005).

In contrast to the P311 overexpression migration studies, the group of Taylor et al. (2000) reported that P311 was decreased upon hepatocyte growth factor/scatter factor (HGF/SF) c-MET induced migration in a human leiomyosarcoma cell line (SK-LMS). The cells became metastatic and obtained increased tumorigenic capacities (Jeffers et al., 1996; Taylor et al., 2000). The fact that migration of these cells was driven by c-Met was not out of the ordinary, since it was already shown that both the mesenchymal and amoeboidal migration pathways in carcinoma cells are stimulated by the c-Met pathway (Huang et al., 2014; **Figure 2**). While low P311 mRNA levels can be observed in glioma cell lines with a high expression of Met-HGF/SF, a tumor suppressive function for P311 was excluded since P311 overexpression in U118 glioma cells did not interfere with tumor growth *in vivo*. This suggested that diminished P311 expression is the result of a more transformed and tumorigenic phenotype. A similar observation was made for terminally differentiated human cortical neuronal cells (HCN-1A and HCN-2) where nerve growth factor exposure decreased P311 expression (Taylor et al., 2000).

During fibrosis, organ resident cells transform into myofibroblasts and accumulate at the site of injury due to chemokines. Interfering with P311 expression or function and thus with the migration potential of these myofibroblasts could be an opportunity to dampen scar formation. However, one needs to consider whether this would not lead to an accumulation of “triggered or activated” myofibroblast far from the injury site which might not be able to contribute to the resolution of the fibrosis once the injury subsides.

## P311 Stimulates Regeneration and Differentiation Toward a Myogenic Phenotype

**FIGURE 4:**
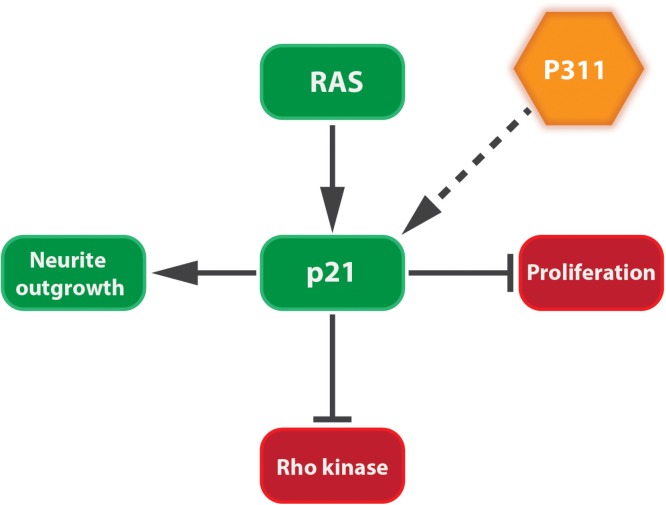
P311 induces the expression of p21^Waf1^. p21^Waf1^ stimulates neurite outgrowth by blocking cell proliferation and Rho kinase. This is stimulated by both Ras signaling and P311, although it is not yet known if the latter goes directly or indirectly.

Tissue development and regeneration, at least in brain, muscle, and lung, can be influenced by P311 expression (Studler et al., 1993; Fujitani et al., 2004; Siller-Lopez et al., 2004; Ooi et al., 2006). The differentiation of a human neuroblastoma cell line (RTBM1), induced by retinoic acid, was accompanied by an increased P311 expression and neurite outgrowth (Ueda, 2001). This is not surprising, since these probing extensions migrate toward the neighboring cells using lamellipodia and filopodia, mediated by the RAC1 pathway (Alberts et al., 2008). Upon neuron axotomy in adult rats, P311 is transiently upregulated between day 3 and day 21 post-surgery (Fujitani et al., 2004). Overexpressing P311 in undifferentiated PC12 cells (rat adrenal gland) or differentiated dorsal root ganglions induced neurite outgrowth, due to the induction of cyclin-dependent kinase inhibitor p21^waf1^. During neuronal differentiation p21^waf1^ is up-regulated to prevent cells from entering the cell cycle (Billon et al., 1996; van Grunsven et al., 1996) and can block Rho kinase activity leading to neurite outgrowth (Erhardt and Pittman, 1998; Olson et al., 1998; Yamashita et al., 1999; Tanaka et al., 2002; Fujitani et al., 2004; Tanaka et al., 2004; **Figure 4**). Moreover, facial neurons that were transfected *in vivo* with P311 cDNA regenerated almost three times as fast as non-transfected facial neurons. This could suggest that P311 can interfere with Rho signaling by inducing p21^waf1^ expression through a thus far unknown mechanism (Fujitani et al., 2004). During human and mouse lung morphogenesis, P311 expression peaks during the saccular and alveolar formation. Smokers who develop emphysema express less P311 compared to smokers without emphysema. Mouse pups treated with dexamethasone, an inhibitor of alveolization, showed a decreased P311 expression when compared to the saline-treated littermates (Zhao et al., 2006). Together this suggests that P311 seems to be involved in the alveolar repair upon injury.

In muscular tissue, P311 expression increases during embryonic pig development and stays active postnatally (Ooi et al., 2006). The opposite was demonstrated for muscle atrophy by two independent studies, one on rats and one on piglets, both looking for molecular patterns that occur during muscle atrophy. Muscle wasting due to skeletal muscle atrophy resulted in a decreased P311 expression, together with other muscle growth stimulating genes, and an increase in E3 ubiquitin ligase enzyme (MAFbx) (Lecker et al., 2004; Ooi et al., 2006). An artificial induction of P311 expression in fibroblasts (3T3 and C3H10) and undifferentiated muscle cell lines (C2C12) on the other hand, induces the expression of muscle-specific transcription factors like myogenic differentiation 1, serum responsive factor (SRF), and myosin heavy chain 4, but less of smooth or skeletal muscle-specific genes. The proliferation rate of these cells increased, but differentiation toward myotubes was attenuated. Myogenic factor 5 (Myf5) was downregulated in all studies, which can be explained by the fact that cell proliferation inhibits Myf5 (Lindon et al., 1998; Pan et al., 2002; Ooi et al., 2006). One of the most important genes that was upregulated is SRF, which interacts with its cofactor myosin light chain kinase 1 to bind to the alpha-smooth muscle actin (α-SMA) promoter in response to matrix stiffness and regulates the expression of other pro-fibrotic genes after TGFβ1 stimulation (Hirschi et al., 2002; Huang et al., 2012). Furthermore, it was shown that SRF has many target genes involved in cytoskeletal organization, migration, and cell proliferation, suggesting that this might be one of P311’s key targets (Soulez et al., 1996; Schratt et al., 2002; Miano et al., 2007; Nolte et al., 2013). Similar to cortical neuron differentiation, when terminal differentiation was induced in C2C12 cells by the broad spectrum inducer of muscle differentiation calcineurin, P311 expression was reduced and accompanied by less cell proliferation (Ooi et al., 2006; Bassel-Duby and Olson, 2003). A correlation between P311 levels and cell proliferation is also described in hepatic stellate cells, invasive glioma cells, and sensory epithelia from the inner ear. Knocking down P311 in hepatic stellate cells reduced cell proliferation *in vitro*. In glioma cells on the other hand, overexpression of anti-tumorigenic gene, melanoma antigen family D1 (DLXIN-1), resulted in less proliferation due to less matrix metalloproteinases (MMP)2 and 9 activity and through direct interaction with P311, DLXIN-1 blocks P311’s invasive function. Finally, P311 is more expressed in regenerating sensory hair cells compared to hair cells that do not renew (Hawkins et al., 2006; Reddy et al., 2011; Guimaraes et al., 2015).

## The P311–TGFβ1 Paradox

The TGFβ1 pathway is one of the main pathways involved in tissue fibrosis. TGFβ1 is a pro-fibrogenic agent, whose precursor [TGFβ bound to a latency-associated protein (LAP)] is stored in the matrix and its activated by proteolytic cleavage by MMP-2, MMP-9, or thrombospondin-1 or mechanical cleavage by integrins, upon tissue damage (Leask and Abraham, 2004; Liu et al., 2009a). Active TGFβ1 accumulates and stimulates an epithelial-to-mesenchymal transition (EMT)-like conversion, and the conversion of fibroblasts and mesenchymal cells into myofibroblasts (Leask and Abraham, 2004; Desmouliere et al., 2005; Breitkopf et al., 2006; Bae et al., 2012). Upon stimulation, TGFβ1 binds to the heteromeric TGFβ1 receptor followed by signaling to TGFβ receptor I kinase, which phosphorylates the receptor-activated SMAD family members, SMAD2 and 3, intracellularly. Phosphorylated SMAD2/3 binds to SMAD4, after which the complex translocates to the nucleus, where the complex binds to p300 and stimulates the transcription of collagen and *Acta*2, as well as other factors involved in cell proliferation and differentiation of mesenchymal cells (Heldin et al., 1997; Leask and Abraham, 2004; Breitkopf et al., 2006; Bae et al., 2012). Apart from its role in migration and proliferation, P311 has been strongly associated with the TGFβ1 pathway.

The mechanisms by which P311 can control the TGFβ1 pathway are numerous and depending on the experimental setup it can be stimulatory or inhibitory. P311-overexpressing 3T3 cells, which do not express endogenous P311, can differentiate into myofibroblasts but have inactive TGFβ proteins. In these cells P311 binds to LAP, rendering TGFβ1 and 2 inactive and preventing auto-induction (Piek et al., 2001; Pan et al., 2002; Paliwal et al., 2004). Consequently, these cells express less TGFβ1 and β2, collagen 1, and MMP 2 and 9, suggesting that P311 might be part of an intrinsic anti-fibrotic mechanism in myofibroblasts (Pan et al., 2002). In a renal tubular epithelial cell line (NRK-52E) P311 can inhibit EMT. TGFβ1 treatment stimulates proliferation and can induce EMT in these cells, accompanied with a downregulation of E-cadherin and an α-SMA upregulation. These effects can be blocked by overexpression of P311 (Qi et al., 2015). These data indicate that overexpression of P311 can reverse or prevent EMT of renal tubular cells and 3T3 fibroblasts *in vitro* (Pan et al., 2002; Paliwal et al., 2004). On the other hand, several studies demonstrate that P311 stimulates TGFβ1 and 2 expression in vascular and lung smooth muscle cells (Badri et al., 2013; Yue et al., 2014). P311 null mice suffer from vascular hypotension, due to a shortage in TGFβ1 and β2 proteins and the absence of P311 in vascular smooth muscle cells results in less Rho A activity and consequently less contractility (Loirand and Pacaud, 2010). In these P311 null mice there is a striking discrepancy between TGFβ protein levels and mRNA levels, i.e., high *Tgf*β*1/2* mRNA levels but low protein levels, suggesting that P311 is involved in the translation of *Tgf*β*1/2*. Mice overexpressing P311 on the other hand are hypertensive. This was confirmed in samples from normo- and hypertensive patients. P311, and consequently TGFβ protein levels, are highly expressed in tissues of hypertensive patients when compared to healthy ones (Badri et al., 2013).

P311 also appears to be involved in the formation of hypertrophic scar, which is the result of an imbalanced wound healing and is a fibrosis-like process (Singer and Clark, 1999). P311 is upregulated and co-expressed with α-SMA, collagen 1, and TGFβ1 in the scar tissue, while there is no expression detected in healthy skin. P311 overexpression in healthy skin induces the expression of TGFβ1 and increases cell proliferation, while interfering with P311 in fibroblasts derived from scar tissue reduces TGFβ1 expression and cell contractility (Tan et al., 2010). In P311 knockout mice, scars from excision wounds showed reduced TGFβ1-3 protein levels, less collagen deposition, and consequently reduced tensile strength and scar stiffness (Cheng et al., 2017). Increased P311 expression can also be detected in tubular epithelial cells from patients with chronic kidney disease, colocalized and correlated with TGFβ1, while healthy kidney tissue does not express either P311 or TGFβ1 (Wang et al., 2010; Yao et al., 2015). The latter observation is confirmed *in vivo* in mice after an unilateral ureteral obstruction, where P311 protein seems to be colocalized with α-SMA and TGFβ1 in acidophilic degeneration regions. Unilateral ureteral obstruction in P311 null mice results in lower levels of collagen type 1 and α-SMA, a reduced expression of TGFβ receptor 1 and 2 (TGFβR1 and 2), and reduced phosphorylation of SMAD2/3. As indicated in earlier reports, *Tgf*β*1* mRNA levels in these mice did not differ between the wild type and P311 null mice, but they present reduced TGFβ1 protein when P311 was absent (Yao et al., 2015).

**FIGURE 5:**
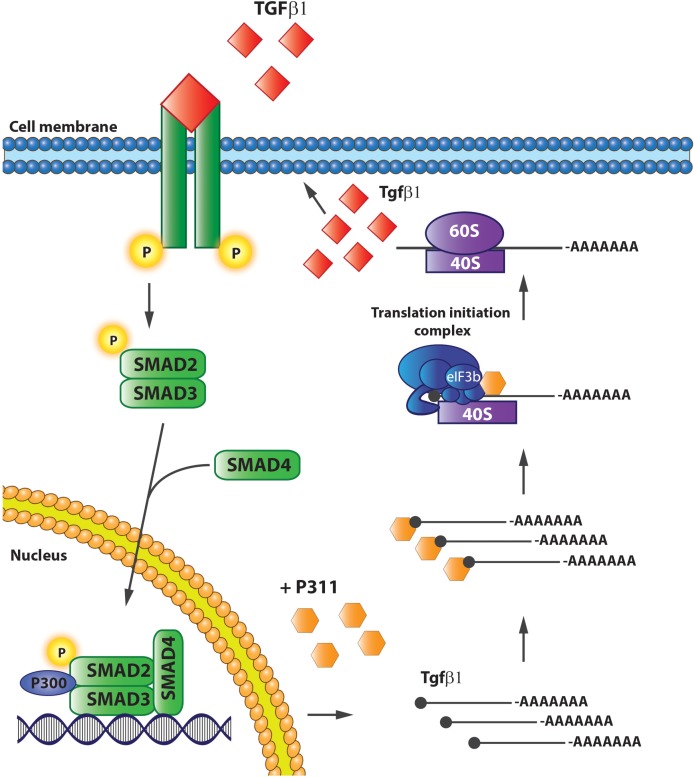
P311 stimulates *Tgf*β*1* translation. P311 binds to the *Tgf*ß*1* mRNA and eukaryotic translation initiation factor 3, facilitating the translation of *Tgf*ß*1* mRNA to protein.

Finally, Yue et al. (2014) gave conclusive evidence that P311 is indeed involved in *Tgf*β*1* translation by showing that it interacts directly with eukaryotic translation initiation factor 3, subunit B (eIF3b), and a protein of the translation initiation complex. In addition, P311 forms a complex with the 5′UTRs of *Tgf*β*1*, 2, and 3 by which it recruits *Tgf*β mRNA and stimulates its translation (Yue et al., 2014). 3T3 fibroblasts without P311 do translate *Tgf*β*1*, but an exogenous expression of P311 can increase the levels of total and active TGFβ1 proteins, which is accompanied with increased mRNA levels due to auto-induction (**Figure 5**). Finally, they also showed that the interaction between P311 and eIF3b occurs on the P311–eIF3b binding motif, which is a highly conserved sequence among different eukaryotes (Yue et al., 2014). These results were confirmed in a study on skin re-epithelialization, where they indicate P311 as a driver of epidermal to mesenchymal transition upon skin damage. Epidermal stem cells expressing exogenous P311 had increased active and LAP-bound TGFβ1 protein levels, increased expression of *Tgf*β*R1* and 2 mRNA, and increased SMAD2/3 phosphorylation, while gaining mesenchymal features. The P311-induced transition was blocked when TGFβRI/II kinases were inhibited. Furthermore, in these cells P311-enhanced methylation of the *Tgf*β promotor and an increased 5′ and 3′ UTR luciferase activity was observed, indicating that P311 stimulates *Tgf*β mRNA transcription (Li et al., 2016).

Although contradictory results exist, it seems that there is a clear correlation between P311 and modulation of TGFβ1 protein levels and its signaling pathways; *in vivo* data indicates that P311 stimulates the TGFβ1 pathway, this was confirmed in cultured primary cells and cell lines (Tan et al., 2010; Wang et al., 2010; Badri et al., 2013; Yue et al., 2014; Yao et al., 2015) but contradicted in two cell lines (Pan et al., 2002; Paliwal et al., 2004; Qi et al., 2015). Nevertheless, some plausible mechanisms have been suggested to explain the observed *in vivo* events, indicating that P311 is an upstream regulator of the TGFβ1 pathway (Yue et al., 2014; Li et al., 2016).

## P311 as a Therapeutic Target?

Currently, different strategies are being applied in clinics/clinical trials to treat tissue fibrosis; anti-fibrotic, anti-inflammatory, anti-oxidant therapies, or combinations of the previous (Schuppan and Kim, 2013; Huang et al., 2015; Schon et al., 2016; Wang et al., 2016). Blocking the TGFβ1 pathway with, for example, fresolimumab or lysyl oxidase ligand 2 antibodies, or stimulating the cells with peroxisome proliferator-activated receptor γ agonists like pioglitazone, are both anti-fibrotic strategies (Rice et al., 2015; Wang et al., 2016). Interferon γ and Ribavirin on the other hand are administered to reduce inflammation (Fernandez et al., 2006), while vitamin E and anthocyanin are two anti-oxidation compounds (Sanyal et al., 2010; Wang et al., 2016). These compounds work well to reduce fibrosis, but are, however, hampered by unwanted side effects (Schon et al., 2016). Targets with a wide range of effects like TGFβ1 or interferon inhibition are supposed to be very efficient to reduce fibrosis, but since they are not tissue-specific, they come with systemic complications. Subjects suffer from mild adverse side effects like anemia, depression, rash, fever, myalgia, internal bleeding, and sometimes even major side effects like worsening of the fibrosis or developing cutaneous neoplasms (Fernandez et al., 2006; Lacouture et al., 2015; Rice et al., 2015). A therapeutic target involved specifically in fibrosis development is needed, preferably combined with a precise delivery method (Gandhi, 2017).

**FIGURE 6:**
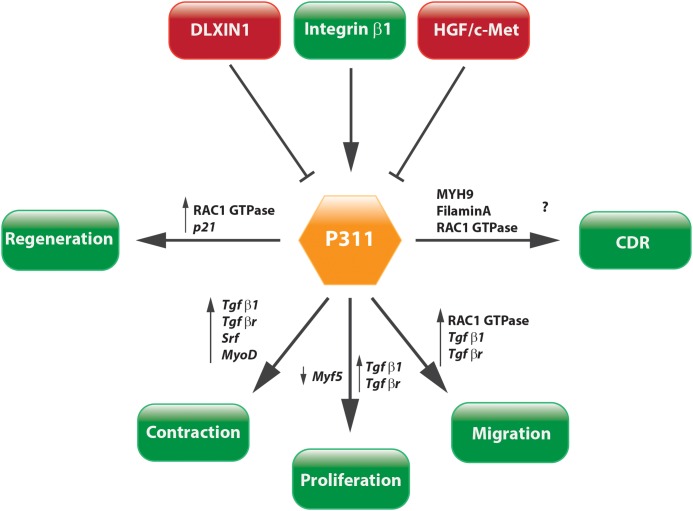
Concluding scheme. P311 is involved in a wide range of pathways. Most importantly, it stimulates cell proliferation, migration, and contraction, which are core characteristics of tissue fibrosis.

The question whether P311 is an interesting therapeutic target to be considered for anti-fibrotic therapy still remains unanswered, but circumstantial evidence indicates it might be a good candidate. Tissue fibrosis is mainly characterized by proliferative and contractile resident myofibroblasts and myofibroblast-like cells (Friedman et al., 2013; Darby et al., 2014). A process in which TGFβ1 is a master regulator that initiates a self-sustaining loop of attracting and activating myofibroblasts (Ignotz and Massague, 1987; Munger et al., 1999; Li et al., 2007; Giacomini et al., 2012). Studies provided evidence that P311 plays a role in different processes that are initiated during fibrosis development and stimulates the translation of *Tgf*β*1* mRNA (**Figures 5**, **6**). Moreover, in P311 null mice, the TGFβ1 pathway is clearly less active, which results in hypotensive mice and reduces kidney fibrosis (Badri et al., 2013; Yao et al., 2015). With regard to organs like liver and lung, there is not yet much known regarding the interplay of P311 and TGFβ1 pathways (**Table 1**). It was demonstrated that P311 could induce a myogenic phenotype in fibroblasts and stimulate its migration (Taylor et al., 2000; Pan et al., 2002; McDonough et al., 2005; Shi et al., 2006; Guimaraes et al., 2015; **Figure 6**). Finally, P311 might have a role in integrin β1 recycling, since P311 interacts with Filamin A and MYH9, while the former is an interconnecting protein between F-actin and integrin β1, the latter is responsible for integrin β1 recycling with F-actin, which is stimulated by the RAC1 pathway (McDonough et al., 2005; Huang et al., 2009; Liu et al., 2011; Casalou et al., 2014; Yue et al., 2014; **Figures 3**, **6**). Silencing P311 *in vivo* or interference with P311 functionality might hamper the fibrogenic process and could allow the tissue to enter the regeneration phase.

**Table 1:** Available data on the role of P311 in kidney, liver, and lung.

Organ	Kidney	Liver	Lung
Healthy tissue	• Low overall P311 expression human (Wang et al., 2010; Yao et al., 2015)	• Low overall P311 expression mouse (Guimaraes et al., 2015)	No data available
*In vivo* studies	• Tubular epithelial cells from injured kidneys express more P311 (Wang et al., 2010; Yao et al., 2015)	• Hepatic stellate cells express more P311 upon liver injury mouse (Guimaraes et al., 2015)	•P311 is important for alveolar development
	• Absence of P311 reduces TGFβ1 and 2, αSMA and collagens production upon injury human (Wang et al., 2010; Yao et al., 2015) and mouse (Yao et al., 2015)		• P311 expression is associated with a lower risk for emphysema development (Zhao et al., 2006)
*In vitro* studies	• In a NRK-52E cell line P311 expression prevents EMT induced by TGFβ1 (Qi et al., 2015)	• In primary hepatic stellate cells P311 knockdown hampers cell migration and reduces cell proliferation (Guimaraes et al., 2015)	• In a human lung smooth muscle cell line P311 stimulates TGFβ1-3 translation (Yue et al., 2014)


The safety of P311 inhibition can be estimated as relatively high, since P311-deficient mice do not present a detrimental phenotype, indicating that development and homeostasis of the body might not be influenced by P311 inhibition. However, since P311 stimulates cell migration, proliferation, and regeneration it indicates that untargeted delivery of siRNA or a compound that could block P311 function *in vivo* might randomly interfere with crucial processes for tissue repair. This can be deduced from studies that showed that patients with less P311 have a decreased alveolar repair after emphysema (Zhao et al., 2006). Therefore, a specific delivery of a P311 siRNA or inhibitory compound to resident myofibroblastic cells in the fibrotic organs would be essential. Local delivery of compounds or siRNA, either free or in nanocomplexes like liposomes, is not always straightforward. While hydrogels and aerosols can achieve this for the treatment of scars or lung fibrosis, respectively (Ivanova et al., 2013; Manosroi et al., 2013; Gumel et al., 2015; Sun, 2017), targeting myofibroblasts in the liver or kidney requires targeted nanomedicine. This can be achieved by using targeted viral vectors (Reetz et al., 2013; Cooney et al., 2018), but over the last decennia a wide array of non-viral delivery systems has been developed for both the delivery of siRNAs and compounds (**Table 2**). For example, delivery of siRNA loaded in targeted liposomes, as already been shown for hepatic and pancreatic fibrosis (Sato et al., 2008; Davies et al., 2014), or an ultrasound microbubble delivery of shRNA to kidneys (Li et al., 2018). Such a specific inhibition of P311 in resident myofibroblasts might interfere with the fibrotic cascade by decreasing cell migration and proliferation and would interfere with the increased TGFβ1 protein levels. Due to this, the accumulation of myofibroblasts at the site of injury might be attenuated and the tissue would be able to initiate resolution of the extracellular matrix.

**Table 2:** Overview of non-viral delivery systems and targeting molecules that are being used for the delivery of compounds and siRNAs to fibrotic organs.

Non-viral delivery systems		

System	Targeted organ	Reference
Lipid-based carriers (including liposomes, niosomes, lipid modified NP, etc.)	Liver, lung, and skin	Togami et al., 2015; Lyon et al., 2017; Blueschke et al., 2018
Gold particles	Liver and skin	Das et al., 2010
Silica particles	Liver and skin	Chang et al., 2005; Das et al., 2010; Morry et al., 2015
Ultrasound microblubbles	Kidney and liver	Zhang et al., 2013; Li et al., 2018

**Targeting molecules**		

**Targeting molecule**	**Membrane target**	**Reference**

Vitamin A	Retinol binding protein	Sato et al., 2008; Ishiwatari et al., 2013; Otsuka et al., 2017
RGD	Collagen type 4 receptor	Du et al., 2007; Chai et al., 2012
PDGF	PDGF receptor	Li et al., 2012
tbFGF	FGF receptor	Togami et al., 2015
Synaptophysin antibody	Synaptophysin	Luli et al., 2016
M6PHSA	Mannose-6-phosphate/insulin-like growth factor receptor	van Beuge et al., 2013


*NP, nanoparticles; RGD, Arg-Gly-Asp peptide; PDGF, platelet-derived growth factor; tbFGF, truncated basic fibroblast growth factor; M6PHSA, mannose-6-phosphate coupled to human serum albumin.*

## Conclusion

A large body of evidence suggests that P311 is necessary for correct TGFβ1 production and its inhibition might result in a less myofibroblastic phenotype, decreased TGFβ1 protein expression and collagen deposition. Importantly, few studies have explored the *in vivo* effects of P311 modulation and its consequence to fibrosis (Yao et al., 2015). Thus, the potential of P311 as an anti-fibrotic target has still to be investigated in other organs, such as lung, liver, and skin, all of which have a high propensity to develop fibrosis. Nevertheless, the possibility of combining specific delivery targeting (i.e., targeted liposomes) and P311 inhibition may prove to be an efficient and safe treatment due to the distinct expression and role of this protein in fibrosis development.

## Author Contributions

LS and LvG conceived the work and provided the layout for it. LS and IM drafted the work. LvG critically revised the text and figures and approved the final manuscript for publication.

## Conflict of Interest Statement

The authors declare that the research was conducted in the absence of any commercial or financial relationships that could be construed as a potential conflict of interest.
